# Comparison of VMAT complexity‐reduction strategies for single‐target cranial radiosurgery with the Eclipse treatment planning system

**DOI:** 10.1002/acm2.13014

**Published:** 2020-09-13

**Authors:** Eric C. Lobb, Michael Degnan

**Affiliations:** ^1^ Department of Radiation Oncology Ascension NE Wisconsin – St. Elizabeth Hospital Appleton WI USA; ^2^ Department of Radiation Oncology The Ohio State University Comprehensive Cancer Center Columbus OH USA

**Keywords:** complexity, radiosurgery, SRS, treatment planning

## Abstract

Complexity in MLC‐based radiosurgery treatment delivery can be characterized by the efficiency of monitor unit (MU) utilization and the average MLC leaf separation distance for a treatment plan. A reduction in plan complexity may be desirable if plan quality is not impacted. In this study, a number of strategies are explored to determine how plan quality is affected by efforts to reduce plan complexity. Ten radiosurgery cases of varying complexity are retrospectively planned using six optimization strategies: an unconstrained volumetric modulated arc therapy (VMAT) technique, a MU‐constrained VMAT technique, three techniques using various strengths of the aperture shape controller (ASC), and a hybrid technique consisting of a final‐stage VMAT optimization applied to a dynamic conformal arc leaf sequence (ODCA). The plans are compared in terms of MU efficiency, MLC leaf‐separation, conformity index (CI), gradient index (GI), and QA measurement results. The five VMAT techniques exhibited only minor differences in CI and GI values, though the ASC and MU‐constrained techniques did require 6–20% fewer MU and had mean field apertures 5–19% larger. On average, the ODCA technique had CI values 3.5% lower and GI values 1.0–2.5% higher than the VMAT techniques, but also had a mean field aperture 24–47% larger and required 16–32% fewer MU. The QA measurement results showed a 0.61% variation in mean per‐field 2%/1 mm gamma passing rates across all techniques (range 96.81%–97.42%), with no observed correlation between passing rate and technique. For simple targets, the ODCA technique achieved CI results that were equivalent to the unconstrained VMAT technique with an average 30% reduction in required MU, an average 50% increase in mean leaf separation distance, and brain V12_Gy_ values within 0.38 cc of the VMAT technique for targets up to approximately 2 cm diameter. For MLC‐based single‐target radiosurgery, plan complexity can often be significantly reduced without an equivalent reduction in plan quality.

## 1 INTRODUCTION

Stereotactic radiosurgery is a widely available technique for the treatment of intracranial tumors and functional abnormalities with a long history of development and refinement.^1^ Radiosurgery utilizes beams which are tightly collimated to the target volume in order to deliver ablative doses in a single fraction or a small number of fractions. Modern radiosurgery may be delivered using a variety of technologies, one of the most recent of which is multileaf collimator‐based radiosurgery using mechanically precise linear accelerators that can achieve overall non‐coplanar isocentric accuracy within 0.6 mm.^2^, ^3^, ^4^, ^5^

MLC‐based radiosurgery delivery with a linear accelerator can be accomplished using a number of techniques.^6^ Static treatment fields with stationary apertures focused on the target from multiple directions utilizing combinations of couch, collimator, and gantry rotation would constitute a basic three‐dimensional (3D) planning and delivery technique. Conformal arc techniques where the gantry rotates during beam delivery are also common. Arcs are generally delivered at multiple couch angles and may utilize either static per‐field MLC apertures or dynamic apertures that conform to the target shape during rotation.

Volumetric modulated arc therapy (VMAT) is a well‐established treatment delivery technique which utilizes simultaneous modulation of the MLC aperture, dose rate, and gantry rotation speed to generate highly conformal dose distributions.^7^, ^8^ For radiosurgery applications, multiple VMAT treatment fields are typically utilized with three or more couch angles to achieve optimal dose conformity and rapid dose falloff at the target edge.

The delivery complexity of VMAT treatment plans is wide‐ranging, and significant efforts have been made to quantify this complexity and investigate how it correlates to deliverability and results of pre‐treatment patient‐specific quality assurance measurements.^9^, ^10^, ^11^, ^12^, ^13^, ^14^, ^15^, ^16^, ^17^ Reduction of delivery complexity for VMAT radiosurgery may be desirable if comparable plan quality can be achieved. In this study, plan complexity refers to the efficiency of monitor unit (MU) utilization and the complexity of MLC leaf sequences utilized with the treatment fields, for which MLC aperture size and leaf separation distances are used as surrogates.

Less complex MLC leaf sequences using larger average aperture sizes may result in better dosimetric agreement between the plan and delivery because of greater similarity between the plan parameters and modeling data, though such an approach must be carefully evaluated to ensure that it does not compromise the sharpness of the dose gradient at the target edge. Pretreatment verification measurements for VMAT radiosurgery plans may be challenging if the treatment fields are comprised of very small highly modulated beamlets, as detector spacing in typical two‐dimensional (2D) or 3D arrays may not be suitable for such measurements. Radiochromic film can be utilized for high‐resolution spatial measurements at the cost of time efficiency, but accurate film dosimetry also has equipment and workflow considerations that must be carefully considered,^18^, ^19^, ^20^, ^21^ and these may include separate point measurements of absolute dose which requires an appropriately sized small‐field dosimeter. Verification measurements using onboard electronic portal imaging devices have significantly improved resolution compared with most independent 2D or 3D arrays as well as high time efficiency, but they do not measure physical dose and do not constitute a “True Composite” verification technique, defined in AAPM TG‐218 as the simulation of treatment delivery to a stationary measurement device placed on the treatment couch using the actual treatment delivery parameters.^22^

Reduction in the plan MUs also has the benefit of reducing scatter and leakage dose to the patient as well as reducing treatment delivery time, although modern flattening filter‐free photon modes support higher dose rates that can reduce this time‐saving benefit. Treatment apertures which are on average more open may also benefit from a reduced contribution of in‐field/ edge‐of‐field interleaf and leaf‐abutment MLC leakage to the total field fluence, components of the beam model which are generally less robust than for the open field.

In this study, a selection of clinical single‐target radiosurgery cases were planned using various VMAT techniques designed to reduce the delivery complexity. The resultant plans were compared in the context of leaf sequence complexity and MU efficiency as well as conformity and gradient indices commonly used in the evaluation of radiosurgery plans. Per‐field and per‐plan quality assurance measurements and analysis was performed for a subset of the treatment plans generated in this study for the purpose of evaluating whether or not patient‐specific quality assurance passing rates were correlated with these delivery complexity metrics. Finally, the goal was to use this data to identify situations, if any, where a standard VMAT optimization technique produces unnecessarily complex treatment plans compared with dosimetrically appropriate alternatives.

## 2 MATERIALS AND METHODS

### 2.A Case selection

Ten initial clinical cases were selected for retrospective planning and evaluation, with those cases being representative of the type and complexity treated in our institution using MLC‐based techniques. In total, there were six intact brain metastasis cases, two postoperative resection cavity cases, and two acoustic neuroma cases. Following an initial analysis of the results, an additional ten cases comprised of intact brain metastasis, simple in shape with no adjacent critical structures, were selected for a targeted analysis between two of the studied techniques. The intent of this targeted analysis was to more robustly investigate a specific clinical scenario that the initial analysis suggested would be most appropriate for a specific complexity reduction strategy. A summary of the cases is provided in Table 1.

**Table 1:** Clinical cases utilized in this study. Note “a” indicates target volume is within 5 mm of an adjacent organ at risk or region of previous treatment; note “b” indicates target volume is within 10 mm of an adjacent organ at risk or region of previous treatment; “c” indicates all organs at risk are separated from the target volume by >10 mm distance. Cases 01–10 were used in the initial analysis, while 11–20 were used in the targeted analysis.

Case #	Type	CTV volume (cc)	Prescription	Note
01	Acoustic neuroma	6.8	2000 cGy/5 Fx	a
02	Metastasis	1.9	2200 cGy/1 Fx	a
03	Resection cavity	21.7	2700 cGy/3 Fx	c
04	Acoustic neuroma	1.1	2000 cGy/5 Fx	a
05	Metastasis	8.5	2700 cGy/3 Fx	b
06	Metastasis	1.5	2700 cGy/3 Fx	a
07	Metastasis	1.2	2400 cGy/3 Fx	a
08	Resection cavity	26.0	2400 cGy/3 Fx	c
09	Metastasis	1.7	2000 cGy/1 Fx	c
10	Metastasis	1.5	2000 cGy/1 Fx	b
11	Metastasis	1.3	2000 cGy/1 Fx	c
12	Metastasis	4.0	2000 cGy/1 Fx	c
13	Metastasis	1.8	2000 cGy/1 Fx	c
14	Metastasis	1.2	2000 cGy/1 Fx	c
15	Metastasis	5.5	2000 cGy/1 Fx	c
16	Metastasis	0.6	2000 cGy/1 Fx	c
17	Metastasis	1.3	2000 cGy/1 Fx	c
18	Metastasis	2.3	2000 cGy/1 Fx	c
19	Metastasis	2.1	2000 cGy/1 Fx	c
20	Metastasis	1.2	2000 cGy/1 Fx	c

### 2.B Plan optimization methods

In total, there were six plan optimization techniques compared within the Varian Eclipse Treatment Planning System (Varian Medical Systems, Inc., Palo Alto, CA), described as follows:

**ODCA (Optimized Dynamic Conformal Arc):** The initial MLC leaf sequence was generated via creation of dynamic conformal arc MLC fields with a 0 or 1 mm PTV aperture margin. The default margin was 1 mm and was changed to 0 mm if the optimization yielded a maximum central target dose of <125%. This DCA leaf sequence was utilized as the starting point for the optimization at the final stage of the multi‐resolution (MR) optimization process (MR4), at which point only minor adjustments to the leaf sequence are permitted. This technique aims to preserve the delivery simplicity inherent in the DCA technique while enhancing dose conformality through the introduction of a minimal degree of aperture modulation.
**VMAT:** Optimization was performed utilizing a standard VMAT technique starting from the first stage of the multi‐resolution optimization process (MR1) with no explicit objective for total number of MUs or aperture shape.
**VMAT_MU:** MR1 VMAT optimization plus an upper total MU limit with maximum strength (100) and a value set to 10% above the total MUs needed in the ODCA plan. This specific MU objective was not extensively studied or determined to necessarily be an optimal value, but was selected based on institutional experience as an objective that would aggressively reduce the plan MU compared to a standard VMAT optimization.
**VMAT_VL:** MR1 VMAT optimization with no MU limit but Aperture Shape Controller set to Very Low
**VMAT_MOD:** MR1 VMAT optimization with no MU limit but Aperture Shape Controller set to Moderate
**VMAT_VH:** MR1 VMAT optimization with no MU limit but Aperture Shape Controller set to Very High

The aperture shape controller (ASC) option is a component of the VMAT leaf sequencer within the photon optimizer (PO) algorithm that penalizes disconnected apertures created by adjacent leaf pairs. The magnitude of the penalty can be adjusted in five discrete steps by adjusting the ASC setting within the Very Low to Very High range, and the higher the ASC setting the more the optimizer will be pushed to join adjacent apertures, theoretically also reducing delivery complexity.

Additionally, a standard non‐optimized dynamic conformal arc treatment plan was generated using a 1mm aperture margin for each case. The set of DCA plans serves as a 3D reference technique and represents a lower limit on delivery complexity for a dynamic treatment plan. In particular, it was anticipated that the DCA technique would have the most efficient MU utilization and the largest overall field apertures while having the least conformal dose distribution, and can therefore serve as a baseline for evaluating how other techniques balance increasing complexity with increasing dosimetric quality. The authors acknowledge that for some of the more challenging clinical cases included in this study, the unmodified DCA plan may not be clinically appropriate due to deficiencies in either dose conformity or adjacent critical structure dose.

Each technique within a given treatment plan utilized an identical set of treatment fields, with all plans utilizing a noncoplanar beam arrangement consisting of either a full or partial arc at the 0° table position along with 2–4 partial arcs at additional table positions selected for optimal field spacing and critical structure avoidance.

**Fig. 1:**
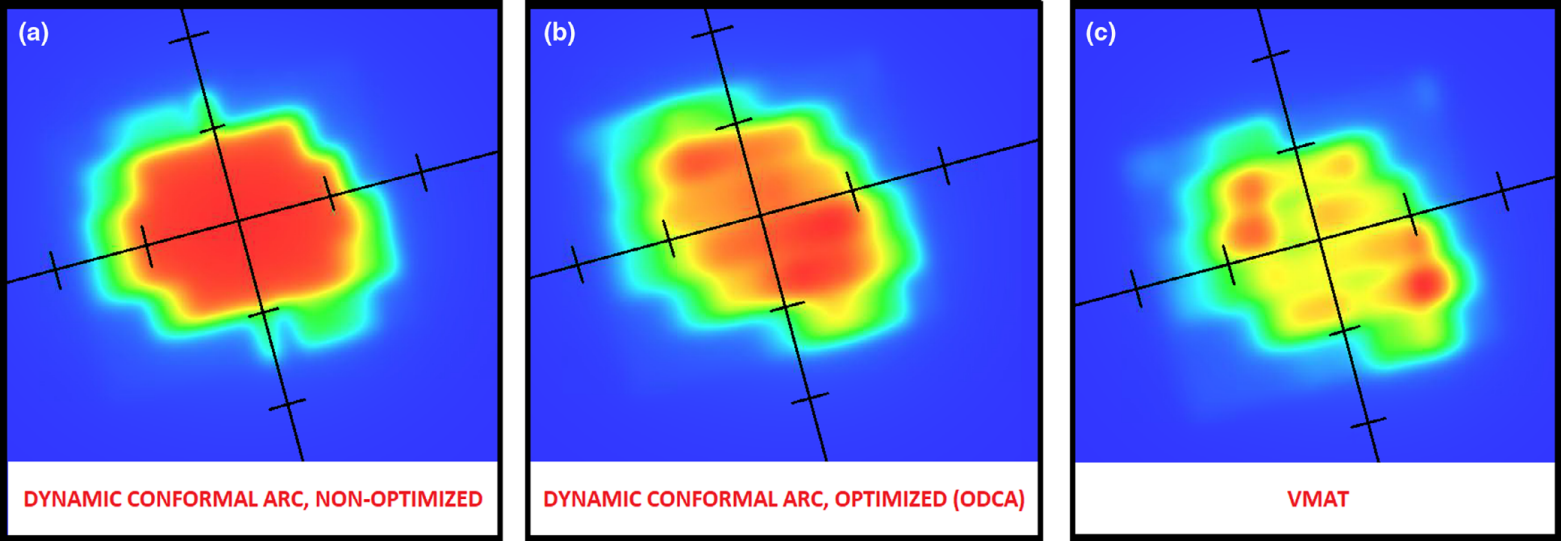
Fluence patterns for the same treatment field generated as a (a) standard dynamic conformal arc field, (b) optimized dynamic conformal arc (ODCA) field, and (c) volumetric modulated arc therapy field. Tick marks on graticule are spaced at 1 cm.

Figure 1 shows typical 2D fluence patterns for the same representative treatment field generated as a standard non‐optimized DCA field, an optimized dynamic conformal arc (ODCA) field, and an unconstrained VMAT field. The standard DCA field shows a nearly homogeneous central fluence with only enough MLC movement to shape the aperture to the target volume during gantry rotation, while the ODCA field introduces a small amount of modulation largely near the field edge. The VMAT technique has a significantly higher degree of aperture modulation both centrally and at the field edge as well as a slight but visibly increased level of in‐field MLC leakage.

### 2.C Dose calculation and optimization parameters

All optimized cases were planned using consistent calculation parameters. The ACUROS v15.5.11 algorithm was utilized with a 1 mm dose calculation grid covering the entirety of the 1 mm slice thickness CT datasets. Optimization was performed using the Photon Optimizer v15.5.11 with structure resolution set to Fine, Convergence Optimization Mode set to On, Dose Calculation Resolution of the optimization engine set to High, and Intermediate Dose performed. The energy utilized for all treatment plans was 6 MV (FF mode).

A common set of optimization objectives based on the unconstrained VMAT optimization served as the starting point for all plans, but objectives were adjusted as needed to meet all coverage and critical structure dose constraints as the mandatory primary objectives while achieving optimal conformity and dose gradient as the secondary objective. Dose gradient and dose conformality were driven by use of the normal tissue objective (NTO) function with a starting dose of 100% at the target edge, a distal isodose level of 30%, a fall‐off factor of 0.35–0.45, and a priority of 250. The use of consistent NTO parameters for all optimized techniques within a given case prevented excessive variation in maximum target dose that could have resulted in gradient index (GI) differences independent of the optimization technique. Within any given case, the largest difference in maximum dose to a volume of 0.03cc between any two optimization techniques was 3.9%.

Following optimization, each plan was normalized such that exactly 95% of the planning target volume received the prescribed dose.

### 2.D Comparison metrics

#### 2.D.1 Leaf separation distance

Average separation between opposed MLC leaves was tabulated per control point for each case and for each planning technique. For simplicity of data presentation, the leaf separations were averaged across all fields within a specific plan and technique to generate a single point of comparison. Only centrally utilized nonclosed leaf pairs were included when computing this metric.

Due to the range of target sizes included in this study, the absolute leaf separation distances covered a wide range that could be difficult to interpret when viewing all the data in aggregate form. To remedy this, all average leaf‐separation data were normalized per‐case to the ODCA result and the normalization factor is included for each case so that the absolute values can be restored if desired.

#### 2.D.2 Monitor unit efficiency

Efficiency of MU utilization was compared by tabulating an MU efficiency ratio for each case and planning technique, defined as the ratio of the total fraction MU and prescribed fraction dose.

#### 2.D.3 Conformity index

Conformity Index (CI) was evaluated using the definition proposed by Paddick et al^23^:

(1) $$CI=\frac{{\left(TV_{\mathrm{PIV}}\right)}^{2}}{TV\times PIV}$$

*TV* is the volume of the target structure, *PIV* is the volume of the prescription dose value, and *TV_PIV_* is the volume of the overlap between the prescription dose value and the target structure. CI values range from 0 (indicating no overlap of the target volume and prescribed dose volume) to 1 (indicating perfect overlap), with values closest to 1 being the most desirable.

#### 2.D.4 Gradient index

GI was evaluated using the definition proposed by Paddick et al^24^:

(2) $$GI=\frac{V_{50\%}}{V_{100\%}}$$

V_50%_ and V_100%_ refer to the volume of the dose clouds created from the 50% and 100% prescription dose values. Radiosurgery planning strongly emphasizes conformity of the prescription dose to the target structure, so lower GI values indicate a shorter falloff distance to the 50% isodose line from the target edge, which is desirable for maximizing the sparing of functional brain tissue surrounding the target structure.

### 2.E Quality assurance measurements

Quality assurance measurements and analysis was performed for the initial set of ten clinical cases using the Varian Portal Dosimetry software, with portal dose prediction generated from the PDIP 15.5 algorithm and portal dose images acquired on an aS‐1000 MV detector at 100 cm source‐imager distance. Immediately prior to each session of quality assurance measurements, the full set of calibrations were performed for the 6 MV “Dosimetry” imaging mode (dark field, flood field, pixel map correction, and dose calibration). All treatment plans were delivered as planned with no modifications other than the removal of couch rotations, which has no impact on the Portal Dosimetry workflow. Gamma analysis was performed per‐field using 2% dose difference and 1mm distance‐to‐agreement input parameters with a 10% low‐dose threshold.

The PDIP algorithm was previously commissioned within our institution specifically for radiosurgery applications as an efficient and high‐resolution tool for verifying patient treatment plans in conjunction with point measurements of absolute dose. The PDIP algorithm and aS‐1000 measurement system has been appropriately validated and extensively utilized within our organization for verification of clinical radiosurgery treatment plans.

## 3 RESULTS

### 3.A Initial case set

#### 3.A.1 Leaf separation distance

Technique comparison results of per‐plan average leaf separation distance values are presented in Table 2. Results for each optimized technique are color‐scaled per‐plan from green to red, corresponding to highest and lowest values, respectively. Results for the standard DCA technique are presented in grey as a 3D technique reference. For 8 of the 10 plans, the ODCA technique resulted in the largest average leaf‐separation distance, with the VMAT_MU technique having the largest values for the remaining two plans. The unconstrained VMAT technique had the smallest values in 8 of 10 cases and for some cases resulted in average leaf separation values that were 30%–50% smaller than complexity‐limited techniques such as ODCA and VMAT_MU.

**Table 2:**
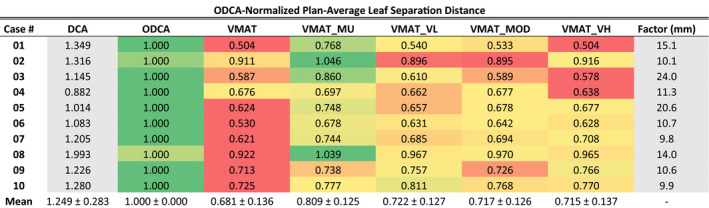
Average leaf separation distances for all techniques and cases, normalized to the optimized dynamic conformal arc (ODCA) technique. Red‐green color scale is case‐specific (rows), with green referring to the largest value and red to the smallest. Multiplying any given result in a row by the Factor in the last column will restore the non‐normalized separation in millimeters. DCA data are presented in grey as a reference 3D technique.

Statistical analysis on the non‐normalized data using a paired *t*‐test at the 95% confidence level revealed that ODCA plans utilized significantly larger average leaf separations than any VMAT technique (maximum *P* = 0.002), that the VMAT_MU technique utilized significantly larger average leaf separations than any other VMAT technique (maximum *P* = 0.023), and that there was no statistically significant difference in average leaf separations between any ASC techniques (*P* range 0.351–0.562). The ASC techniques did, however, all use larger mean leaf separations than the unconstrained VMAT technique (*P* range 0.001–0.021).

#### 3.A.2 Monitor unit efficiency

Technique comparison results of MU efficiency are presented in Table 3, with results for each optimized technique color‐scaled per‐plan from green to red, corresponding to lowest and highest values, respectively. Results for the standard DCA technique are presented in grey as a 3D technique reference. The ODCA technique required the least amount of MUs in 8 of the 10 plans, with the VMAT_MU technique being the most efficient in the remaining two plans. Case 06 resulted in a more than 50% difference in MU efficiency between the ODCA and VMAT technique, the greatest for any comparison.

**Table 3:**
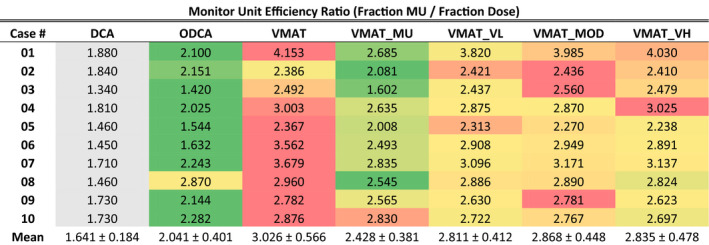
Monitor unit efficiency ratios for all techniques and cases, non‐normalized. Red‐green color scale is case‐specific (rows), with green referring to the smallest value and red to the largest. DCA data are presented in grey as a reference three‐dimensional (3D) technique.

Statistical analysis using a paired t‐test at the 95% confidence level revealed that the ODCA plans required significantly fewer MU than any VMAT technique (maximum *P* = 0.008), the reduction in MU with the VMAT_MU technique was significant compared to all other VMAT techniques (maximum *P* = 0.013), and the increase in MU required for the unconstrained VMAT technique was statistically significant compared to all other VMAT techniques (maximum *P* = 0.030) except for the VMAT_MOD technique (*P* = 0.055). There was no significant difference between any of the ASC techniques, although all ASC techniques did return MU efficiency results that were improved over the unconstrained VMAT technique in a statistically significant way (*P* range 0.017–0.049).

#### 3.A.3 Paddick conformity index

Paddick CI results are presented in Table 4, with optimized plan results globally color‐scaled from red to green, corresponding to 0.75 or lower (red) and a maximum value of 1.0 (green). Results for the standard DCA technique are presented in grey as a 3D technique reference. The only immediately observable trend in the CI data is that the ODCA technique had the lowest CI value in 8 of 10 cases and did not achieve the highest value in any of the cases. The highest per‐case CI value was achieved by the unconstrained VMAT technique in three cases, by the VMAT_MU and VMAT_VL techniques each in one case, by the VMAT_MOD technique in four cases, and by the VMAT_VH technique in two cases.

**Table 4:**
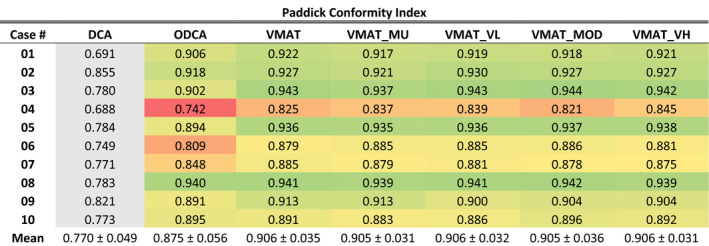
Paddick conformity index values for all techniques and cases, non‐normalized. Red‐green color scale is global, with red assigned to a value of 0.75 or lower and green assigned to a maximum value of 1. DCA data are presented in grey as a reference 3D technique.

Statistical analysis using a paired t‐test at the 95% confidence level revealed that the reduction in CI values for ODCA plans was statistically significant compared to all VMAT techniques (*P* range 0.007–0.021). However, no significant difference was identified between any two VMAT techniques (*P* range 0.442–0.940), including between ASC techniques (*P* range 0.689–0.771).

#### 3.A.4 Gradient index

GI results are presented in Table 5, with optimized plan results globally color‐scaled from green to red, corresponding to 3 or lower (green) and 5 or higher (red). These values correspond to the ideal value (3 or lower) and upper limit value (5) used as planning objectives in our institution. Results for the standard DCA technique are presented in grey as a 3D technique reference. Each optimized technique produced a GI result that was both the best and the worst for at least one clinical case, or within 0.5% of being the best or worst. When looking at the mean technique‐specific value across all cases the constrained and unconstrained VMAT techniques all had results within 1.5% of each other while the ODCA technique on average had a GI value 2.5% higher than the best‐performing unconstrained VMAT technique. For 7 of 10 cases the best and worst GI values were within 5% and no case exceeded a 10% difference.

**Table 5:**
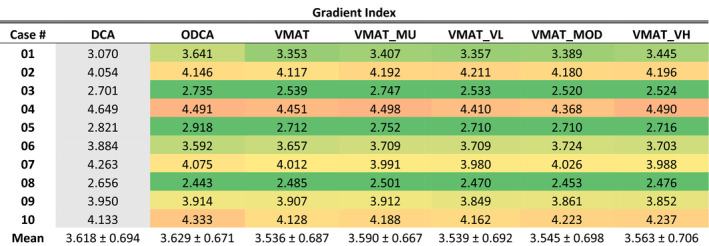
Gradient index values for all techniques and cases, non‐normalized. Green‐red color scale is global, with green assigned to a value of 3 and red assigned to a value of 5. DCA data are presented in grey as a reference three‐dimensional technique.

Statistical analysis using a paired *t*‐test at the 95% confidence level revealed that the increase in GI values for the ODCA and VMAT_MU techniques was statistically significant compared to the unconstrained VMAT technique (*P* = 0.039 and 0.022, respectively), with no significant difference between any other optimized techniques. Specifically, there was no significant difference between any ASC techniques (*P* range 0.098–0.565).

#### 3.A.5 Quality assurance measurements results

**Fig. 2:**
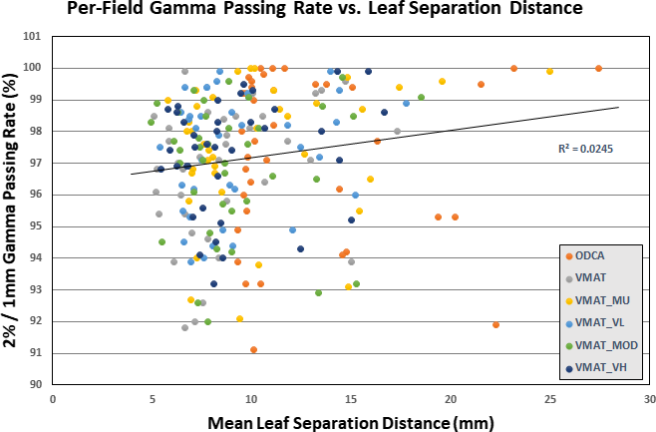
Per‐field gamma passing rate plotted as a function of mean leaf separation distance, with linear trendline.

Mean per‐field gamma passing rates are presented in Table 6, with optimized plan results globally color‐scaled from red to green, corresponding to 93% or lower (red) and a maximum value of 100% (green). Results for the standard DCA technique are presented in grey as a 3D technique reference. Additionally, individual gamma passing rates for each of the 228 optimized treatment fields utilized in this study are plotted against mean leaf separation distance in Fig. 2.

**Table 6:**
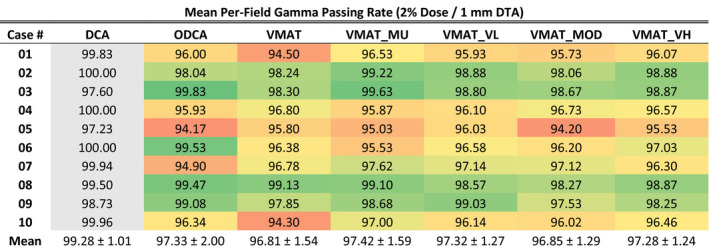
Mean per‐field gamma passing rates for all techniques and cases, non‐normalized. Red‐green color scale is global, with red assigned to a value of 93% or lower and green assigned to a maximum value of 100%. DCA data are presented in grey as a reference three‐dimensional technique.

Statistical analysis using a paired *t*‐test at the 95% confidence level revealed no statistically significant differences in gamma passing rates between any two techniques in the ODCA and VMAT categories (*P* range 0.208–0.496). The increase in gamma passing rates for the non‐optimized 3D DCA plans was statistically significant compared to all other techniques (*P* range 0.000–0.009).

### 3.B Targeted case set

Cases 11–20 were selected to allow for a targeted analysis between the ODCA and unconstrained VMAT techniques for simple ellipsoidal target volumes with no immediately adjacent critical structures. Leaf separation, MU efficiency ratio, GI, and CI results are presented for each case and both techniques in Table 7.

**Table 7:** Metrics for ODCA vs unconstrained VMAT targeted analysis of cases 11–20, comprised of simple ellipsoidal target volumes.

ODCA vs VMAT — Metrics for simple metastasis plans									
Case #	Normalized leaf separation		MU efficiency ratio		Gradient index		Conformity index		Leaf separation factor (mm)
	ODCA	VMAT	ODCA	VMAT	ODCA	VMAT	ODCA	VMAT	
11	1.000	0.719	2.300	3.050	4.286	4.060	0.930	0.930	10.9
12	1.000	0.615	1.860	2.730	3.093	2.952	0.944	0.948	14.7
13	1.000	0.652	2.170	2.790	3.734	3.658	0.941	0.945	10.3
14	1.000	0.702	1.990	2.610	4.260	4.082	0.916	0.926	9.9
15	1.000	0.627	1.800	2.750	2.811	2.732	0.929	0.933	15.8
16	1.000	0.756	2.410	2.860	4.936	4.956	0.914	0.902	8.1
17	1.000	0.643	2.180	3.390	4.140	3.952	0.920	0.925	10.8
18	1.000	0.612	2.200	3.660	3.432	3.363	0.943	0.930	10.7
19	1.000	0.606	1.850	2.810	3.605	3.562	0.937	0.932	10.3
20	1.000	0.653	2.150	3.250	4.206	4.104	0.894	0.906	10.0
Mean:	1.000	0.658 ± 0.048	2.091 ± 0.195	2.990 ± 0.322	3.850 ± 0.604	3.742 ± 0.608	0.927 ± 0.015	0.928 ± 0.014	—

Statistically, there was no difference in CI between the ODCA and VMAT techniques at the 95% confidence level (*P* = 0.745), while the ODCA technique had an average 2.8% increase in GI values that was determined to be statistically significant (*P* < 0.001). The effect of this GI difference on the V12_Gy_ values for the brain tissue surrounding the target volumes is an average of 0.18 cc across all 10 cases, with a maximum increase of 0.38 cc for the ODCA plan in Case 15, which has the largest tumor volume in this additional set of cases (equivalent sphere diameter of 2.2 cm).

There was an average reduction in MU of 30% with the ODCA technique compared to the VMAT technique (*P* < 0.001), corresponding to a nearly 1800 MU average reduction for this set of cases with a single‐fraction dose of 2000 cGy. Also significant was the more than 50% average increase in mean leaf separation values for the ODCA technique relative to the unconstrained VMAT technique (*P* < 0.001).

## 4 DISCUSSION

### 4.A Aggregate data discussion for initial cases

The VMAT_MU and ASC techniques all systematically generated plans that used larger mean leaf separation distances and fewer MUs to deliver the same fraction dose compared to the unconstrained VMAT technique. Normalized leaf separation distances were within 1% of each other for all ASC techniques (range 0.715–0.722), approximately 5% larger than the unconstrained VMAT mean leaf separation distance (0.681). Similarly, the average MU efficiency ratios for the ASC techniques had only a 2% spread (2.811–2.868), requiring approximately 6% fewer MUs than the unconstrained VMAT technique. Interestingly, the VMAT_VL, VMAT_MOD, and VMAT_VH plans which utilized the ASC did not as a rule utilize larger average beamlets or require fewer MU as the strength of the ASC increased. For 6 of the 10 plans, the strongest application of the ASC (VMAT_VH) yielded smaller average beamlet sizes than the weakest application (VMAT_VL).

Prior to data collection, it was hypothesized that larger target volumes such as the resection cavities may be more sensitive to changes in the ASC parameter due to having a wider range of beamlet sizes to apply the optimization penalty to, but the results do not support this. Instead, it is likely that the heightened challenge of meeting volumetric constraints on the surrounding healthy brain tissue with larger‐sized targets dominated the optimization and forced the use of consistently small and potentially disjointed beam apertures, regardless of ASC setting. For the small radiosurgery target volumes, the low number of leaf pairs used in any given treatment field may simply not allow for the generation of enough disjointed apertures for the ASC to have a significant effect.

The VMAT_MU technique utilized leaf separation distances (0.809) approximately 19% larger than the unconstrained VMAT technique (0.681) and required approximately 20% fewer MUs than the unconstrained VMAT technique for this group of cases. Overall, for the leaf separation and MU efficiency complexity reduction metrics the VMAT_MU technique was superior to both the ASC and unconstrained VMAT techniques, and this difference was found to be statistically significant.

For all VMAT techniques, there was no statistically significant difference in mean conformity of dose (CI range 0.905–0.906) and a spread of only 1.5% in GI values between the best and worst performing techniques (range 3.536–3.590). Together with the complexity reduction results previously mentioned, this indicates that, on average, utilizing strict MU limitations during the typical VMAT optimization workflow can result in less complex plans without sacrificing dosimetric quality.

In the initial set of cases, the ODCA technique had an average of 47% larger mean leaf separation distances and required 32% fewer MUs than the unconstrained VMAT technique. Compared to the ASC techniques, these results were 39% larger and 28% fewer, respectively. Finally, compared to the VMAT_MU technique, these results were 24% larger and 16% fewer, respectively. Overall, the ODCA is superior to all VMAT‐based techniques in terms of leaf separation distance and MU efficiency, with all improvements being statistically significant.

In the initial set of cases (01–10), CI in the ODCA plans was lower compared to the VMAT techniques, with an aggregate mean CI value 3.5% lower than the VMAT plans (0.875 vs 0.906), a statistically significant reduction compared to all other optimized techniques. In terms of GI, although ODCA was the best performing technique for Case 06 and Case 08, the mean overall GI values for the ODCA technique were 1.0%–2.5% higher than for the VMAT techniques, although the only statistically significant improvement compared to the ODCA technique was with the unconstrained VMAT technique.

As initially suspected, the standard non‐optimized DCA technique required fewer MU, generally used larger average leaf separations, and exhibited poorer dose conformity than any optimized technique. Overall, introducing a low level of optimization to the DCA plan to form an ODCA plan increased the required MU by approximately 25%, but the per‐case range had significant variation (6%–96%). Mean leaf separation distance was also on average 20% smaller in the ODCA plans than in the standard DCA plans, with the exception of Case 04 where the ODCA technique produced a mean leaf separation distance 13% larger (1.5 mm). This exception is explained by the fact that the ODCA plan in Case 04 was originally generated from a DCA plan with a 0 mm aperture margin, and this initial leaf sequence under‐covered the PTV to an extent that required the ODCA optimization to use larger apertures to meet coverage constraints. CI was uniformly improved in the ODCA plans compared to the standard DCA plans, by an average of 14% (range 7%–31%), a statistically significant improvement (*P* < 0.001). Comparing ODCA and standard DCA GI results, mean values for all plans in aggregate were not statistically different (*P* = 0.897), all results being within 0.5% of one another with exactly half of the cases having lower GI values with the standard DCA technique and the other half having lower values with the ODCA technique.

Radiosurgery cases with mostly spherical targets may be appropriately planned using the non‐optimized DCA technique, but irregularities in target shape or limitations in beam arrangement can result in the need for some manual intervention in beam apertures, sometimes at the per control point level. The ODCA technique is an efficient alternative to these manual adjustments, resulting in improved overall dosimetric quality with only a modest increase in delivery complexity.

The quality assurance measurement results presented in Table 6 and Fig. 2 show only minor variation in mean per‐field gamma passing rates across all cases and optimization techniques, with no statistically significant difference between any two optimized techniques and no observed correlation between passing rate and either optimization technique or aperture size. For optimized techniques, the maximum difference between the highest and lowest gamma passing rate for any given case is 4.0% (Case 06, 99.53% vs 95.53%), while the mean difference between highest and lowest passing rates for all cases is 1.97%. When considering all cases together, the mean passing rate varies by only 0.61% across all optimization techniques (range 96.81%–97.42%). Only 5 of the 60 plans failed to achieve at least 95% of points passing a 2%/1 mm gamma test, and of those cases the lowest result was 94.17% of points passing. Although the improvement is not statistically significant, the VMAT_MU technique did achieve the highest average gamma passing rate of all the techniques and did not contain any of the plans which failed to achieve a minimum 95% gamma passing rate. Of the plans which failed to achieve at least 95% of points passing, two were ODCA plans, two were unconstrained VMAT plans, and one was a VMAT_MOD plan.

The standard non‐optimized DCA technique exhibited higher passing rates nearly uniformly, having the highest per‐field average in 8 of 10 cases. Overall, the standard DCA technique had a passing rate 1.9–2.5 percentage points higher than the optimized techniques, a statistically significant result that is unsurprising considering the delivery simplicity inherent in 3D techniques.

The hypothesis that less complex MLC delivery patterns using larger apertures would result in better agreement between planned and delivered dose does not appear to be supported, at least in the context of verification measurements for *optimized* radiosurgery plans acquired with Portal Dosimetry. The gamma passing rates for ODCA plans was not statistically different from those of VMAT plans, but the decrease in passing rates for ODCA and VMAT plans relative to 3D DCA plans was statistically significant. The introduction of any level of modulation slightly reduced gamma passing rates compared to the 3D plan, but the difference in levels of modulation between optimized techniques did not correspond to an equivalent change in gamma passing rates.

### 4.B Case discussions and targeted analysis

This section discusses a small number of specific cases and associated observations as well as discussion of the targeted analysis between the ODCA and VMAT techniques.

**Fig. 3:**
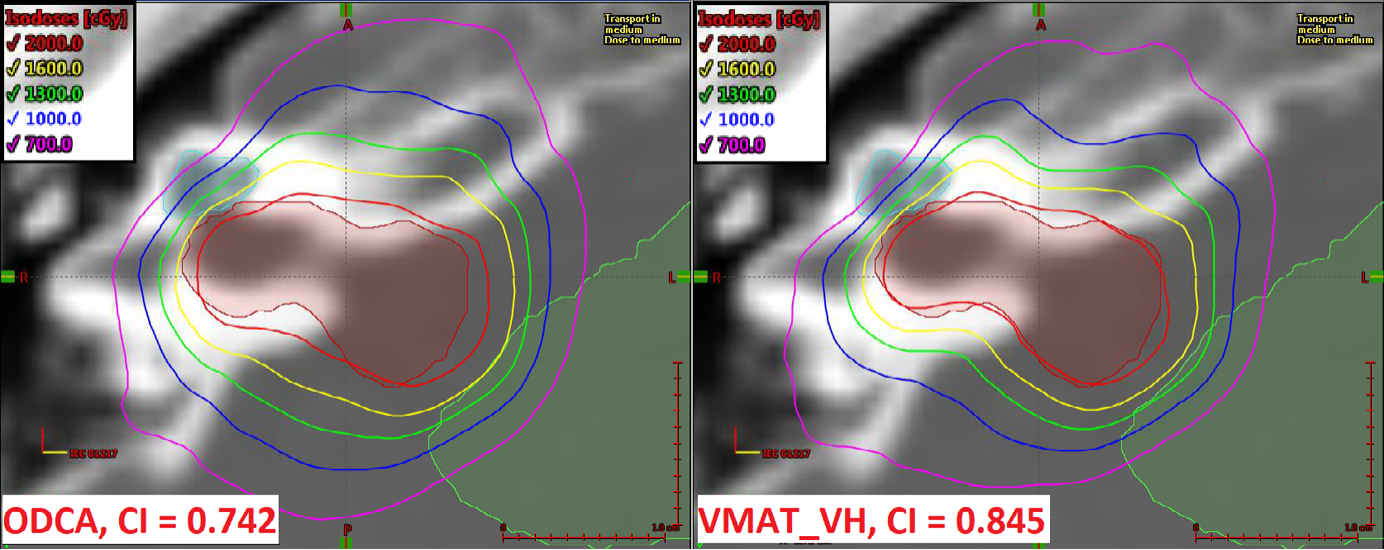
Axial dose comparison for Case 04 (acoustic neuroma, 1.1 cc) showing the optimized dynamic conformal arc technique (left) and VMAT_VH technique (right). Prescription dose is 2000 cGy in five fractions. The full length of the displayed size scale is 1.0 cm in each dimension.

Figure 3 shows an axial dose comparison for Case 04 (acoustic neuroma, 1.1 cc) between the least conformal technique (ODCA, CI = 0.742) and the most conformal technique (VMAT_VH, CI = 0.845). This case had the largest relative difference in CI result between the best and worst performing techniques, and also had the smallest target in the initial set of cases (equivalent sphere diameter of 13 mm) and required dose management to two immediately adjacent critical structures (brainstem and right cochlea) while conforming dose to multiple concave surfaces. Although both techniques met minimum acceptable critical structure dose objectives, the VMAT_VH technique was able to achieve a 13% reduction in maximum dose to the brainstem and a 6% reduction in mean dose to the right cochlea compared to the ODCA technique due to more rapid lateral dose falloff. It is intuitive that such a situation would benefit from the use of a VMAT optimization technique that is not aperture‐constrained to the extent that the ODCA technique is. The specific choice of VMAT optimization technique had only a minor impact on the resulting conformity and GI values, and relevant dose metrics for adjacent critical organs had a spread of <1% across all VMAT techniques. The VMAT_MU technique offered equivalent plan quality to the other VMAT techniques, including the unconstrained VMAT technique, while reducing the required MU by approximately 10%.

**Fig. 4:**
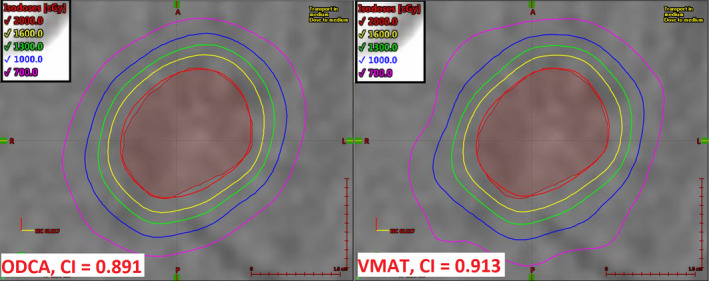
Axial dose comparison for Case 09 (metastasis, 1.7 cc) showing the optimized dynamic conformal arc technique (left) and the unconstrained VMAT technique (right). Prescription dose is 2000 cGy in one fraction. The full length of the displayed size scale is 1.0 cm in each dimension.

Figure 4 shows an axial dose comparison for Case 09 (metastasis, 1.7 cc) between the least conformal technique (ODCA, CI = 0.891) and the most conformal technique (VMAT, CI = 0.913). Case 09 was selected because it is the midpoint in the initial case group in terms of relative difference in CI between the best and worst plans. In this case, the lesion is ellipsoidal with no immediately adjacent critical structures, and qualitative review reveals essentially no difference in the quality of conformality of the prescription isodose surface to the target volume between the techniques which yielded the best and worst CI results. Similarly, the GI values differed between these techniques by <0.2%. However, the ODCA technique utilized 23% fewer MU (1276 MU difference) with a mean aperture size 40% larger than the unconstrained VMAT technique.

For Case 09 and others with similar characteristics (targets without concave surfaces and no abutting critical structures), the ODCA technique achieves a delivery pattern that is less complex with more efficient MU utilization while fully preserving the dosimetric quality of more complex planning techniques. In such cases, there is incentive to explore the more effective complexity‐reduction strategies and compare the results to those achieved with a typical VMAT technique to determine if the additional complexity of the VMAT technique meaningfully enhances the quality of the treatment plan.

Cases 11–20 were selected as examples of such a scenario, and the targeted analysis summarized in Table 7 supports the previous statement. Statistically equivalent CI results can be achieved with approximately 30% fewer MU and 50% larger mean leaf separation distances with the ODCA technique compared to an unconstrained VMAT technique. Although the ODCA technique did have a statistically significant increase in GI values, the practical consequence of this increase was tenths of a cubic centimeter of additional adjacent brain tissue receiving 1200 cGy. The largest target volume in this set of cases (2.2 cm equivalent sphere diameter) had an increase of 0.38 cc in the brain V12_Gy_ metric, a volume that can be represented by an additional 0.25 mm thick rind on a tumor volume of this size.

**Fig. 5:**
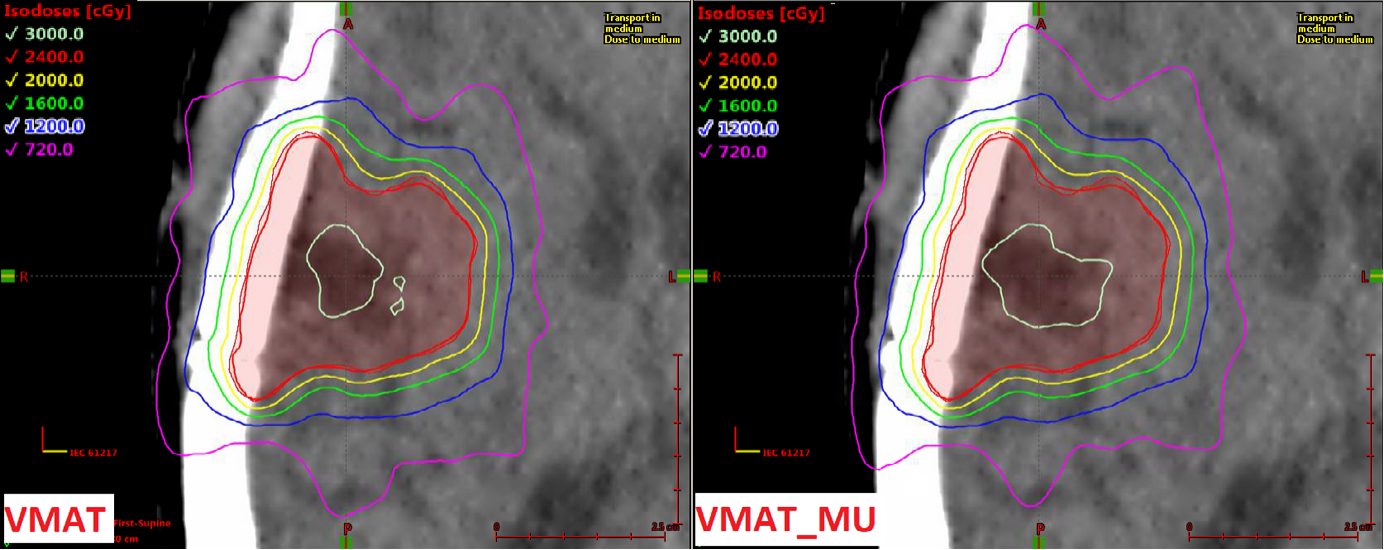
Axial dose comparison for Case 08 (resection cavity, 26.0 cc) showing the unconstrained VMAT technique (left) and the constrained VMAT_MU technique (right). Prescription dose is 2400 cGy in three fractions. The full length of the displayed size scale is 2.5 cm in each dimension.

Figure 5 shows an axial dose comparison for Case 08 (resection cavity with multiple concave surfaces, 26.0 cc) between the unconstrained VMAT and the VMAT_MU techniques. In this case, the mean leaf separation distance was 13% larger in the VMAT_MU technique, the number of fraction MU was 14% fewer (332 MU difference for an 800 cGy fraction dose) in the VMAT_MU technique, and the CI and GI values were within 0.2% and 0.6% of one another, respectively. This is an example of a case where, despite the complexity of shape in the target volume, complexity of the delivery parameters can be reduced with essentially no reduction in overall plan quality.

## 5 CONCLUSIONS

Optimization strategies which aim to reduce the delivery complexity of single‐target radiosurgery treatment plans are largely able to increase mean field aperture dimensions and reduce required MUs while preserving the overall dosimetric quality of the plan. However, certain highly complex target volumes may still benefit from less constrained VMAT optimization approaches in order to achieve optimal dose conformity. Of the VMAT‐based strategies explored here, a simple MU‐constrained optimization appears to best balance delivery complexity and plan quality.

Utilizing a hybrid approach of performing final‐stage VMAT optimization on a simple dynamic conformal arc MLC leaf sequence (ODCA) can significantly reduce the required number of MUs and complexity of the MLC sequence compared to VMAT techniques while improving conformity of dose compared to a non‐optimized dynamic conformal arc plan. Although typically less conformal compared to plans generated with a full VMAT optimization, the difference is not always significant and the reduction in complexity may be favorable, especially for simple ellipsoidal tumor volumes.

High‐resolution quality assurance measurements of each of the 228 treatment fields used across 60 optimized treatment plans did not demonstrate any meaningful correlation between optimization technique or mean aperture size with 2%/1 mm gamma passing rates, indicating that any motivation for reducing complexity in radiosurgery treatment plans should be justified independently from gamma passing rates alone.

Plan quality and plan complexity exist together on a spectrum, but this study demonstrates that complexity can often be significantly reduced without an equivalent reduction in plan quality. Institutions can utilize these and other planning strategies to find the optimal balance for their own clinical cases.

## CONFLICT OF INTEREST

No conflict of interest.
